# Therapeutic targeting of the focal adhesion complex prevents oncogenic TGF-β signaling and metastasis

**DOI:** 10.1186/bcr2360

**Published:** 2009-09-09

**Authors:** Michael K Wendt, William P Schiemann

**Affiliations:** 1 Department of Pharmacology, University of Colorado Denver, Anschutz Medical Campus, Aurora, Colorado 80045, USA

## Abstract

**Introduction:**

Mammary tumorigenesis is associated with the increased expression of several proteins in the focal adhesion complex, including focal adhesion kinase (FAK) and various integrins. Aberrant expression of these molecules occurs concomitant with the conversion of TGF-β function from a tumor suppressor to a tumor promoter. We previously showed that interaction between β_3 _integrin and TβR-II facilitates TGF-β-mediated oncogenic signaling, epithelial-mesenchymal transition (EMT), and metastasis. However, the molecular mechanisms by which the focal adhesion complex contributes to β_3 _integrin:TβR-II signaling and the oncogenic conversion of TGF-β remain poorly understood.

**Methods:**

FAK expression and activity were inhibited in normal and malignant mammary epithelial cells (MECs) either genetically by using lentiviral-mediated delivery of shRNAs against FAK, or pharmacologically through *in vitro *and *in vivo *use of the FAK inhibitors, PF-562271 and PF-573228. Altered Smad2/3 and p38 MAPK activation, migration, EMT, and invasion in response to TGF-β_1 _were monitored in FAK-manipulated cells. TβR-II expression was increased in metastatic breast cancer cells by retroviral transduction, and the metastasis of FAK- and TβR-II-manipulated tumors was monitored by using bioluminescent imaging.

**Results:**

TGF-β stimulation of MECs stabilized and activated FAK in a β_3 _integrin- and Src-dependent manner. Furthermore, by using the human MCF10A breast cancer progression model, we showed that increased FAK expression in metastatic breast cancer cells mirrored the acquisition of enhanced activation of p38 MAPK by TGF-β. Administering FAK inhibitors or rendering metastatic breast cancer cells FAK deficient abrogated the interaction between β_3 _integrin and TβR-II, thereby preventing TGF-β from (a) activating p38 MAPK; (b) stimulating MEC invasion, migration, and EMT; and (c) inducing early primary tumor dissemination to the lungs. Finally, in contrast to FAK depletion, adjuvant FAK chemotherapy of mammary tumors decreased their growth in part by diminished macrophage tumor infiltration.

**Conclusions:**

Our studies identify an essential function for FAK in mediating the interaction between β_3 _integrin and TβR-II, and thus in facilitating the oncogenic conversion of TGF-β required for mammary tumor metastasis. Furthermore, this study establishes chemotherapeutic targeting of FAK as an effective, two-pronged approach in preventing tumor progression both by decreasing innate immune cell infiltration, and by inhibiting early TGF-β-dependent metastasis.

## Introduction

Invasion and metastasis are the most lethal characteristics of breast cancer [1,2]. Transforming growth factor (TGF)-β is a powerful suppressor of mammary tumorigenesis through its ability to repress mammary epithelial cell (MEC) proliferation, as well as through its creation of cellular microenvironments that inhibit MEC motility, invasion, and metastasis. During breast cancer progression, the tumor-suppressing function of TGF-β is frequently subverted, thus transforming TGF-β from a suppressor of breast cancer formation to a promoter of its growth and metastasis [2-4]. Indeed, how TGF-β both suppresses and promotes tumorigenesis remains an unknown and fundamental question that directly affects the ability of science and medicine to target effectively the TGF-β signaling system during the treatment of human malignancies. Deciphering this paradox remains the most important question concerning the biologic and pathologic actions of this multifunctional cytokine [5].

FAK is a ubiquitously expressed protein tyrosine kinase (PTK) whose amino acid sequence is about 90% homologous between humans, chickens, mice, and frogs [6]. An essential function for FAK during mammalian development is evident in the lethality of FAK-deficient embryos at E8.5 [7], presumably due to an indispensable role of FAK in regulating cell migration [8], proliferation, and survival [9]. Along these lines, aberrant FAK expression or activity also supports carcinoma cell metastasis by enhancing these same cellular processes in cancer cells [10], and possibly in cancer stem cells [11], to support tumor angiogenesis [12]. Although it remains to be determined whether altered expression or subcellular localization of FAK possesses true prognostic value to cancer patients, recent studies do provide strong evidence associating increased FAK expression with the development and progression of mammary carcinomas [10,12-15]. To this end, small-molecule inhibitors of FAK have recently been developed and show potent efficacy to inhibit FAK PTK activity specifically, as well as to decrease the growth of subcutaneous tumor xenografts [13,16]. Despite these recent advances, the oncogenic signaling modules targeted by aberrant FAK expression and activity in developing and progressing breast cancers, and their potential role in regulating the activity and composition of associated tumor stroma remain to be fully defined.

We recently identified a critical α_v_β_3 _integrin:TβR-II:Src:Grb2 signaling axis that mediates TGF-β stimulation of MAP kinases in normal and malignant MECs, leading to their acquisition of epithelial-mesenchymal transition (EMT), invasive, and metastatic phenotypes both *in vitro *and *in vivo*. Activation of this oncogenic signaling axis by TGF-β requires β_3 _integrin to form complexes with TβR-II [17-19]; however, whether the β_3 _integrin/TβR-II interaction is direct or mediated *via *an accessory protein remains unknown. The present study addresses this important question, as well as establishes the therapeutic effectiveness of inhibiting FAK PTK activity in a TGF-β-driven model of breast cancer metastasis.

## Materials and methods

### Cell lines and reagents

Normal murine NMuMG and metastatic 4T1 cells were obtained from ATCC (Manassas, VA) and cultured as described previously [18]. 4T1 cells were engineered to express stably firefly luciferase by their transfection with pNifty-CMV-luciferase [20] and selection with Zeocin (500 μg/ml; Invitrogen, Carlsbad, CA). NMuMG cells expressing WT or the nonfunctional mutant D119A-β_3 _integrin were constructed by retroviral transduction, as described previously [19]. The MCF10A cell derivates T1k, Ca1h, and Ca1a were cultured in DMEM/F12 supplemented with 5% horse serum. The construction of NMuMG and 4T1 cells lacking FAK was accomplished by their lentiviral-mediated transduction with a scrambled (*i.e*., nonsilencing shRNA) or verified FAK-specific shRNA sequence encoded in pLentilox3.7-puro [15]. In brief, human 293T cells were transiently transfected with lentiviral packaging vectors (*i.e*., pMD2.G, pRRE, pRSV, and pLentiLox 3.7) according to standard protocols [21], and 48 h after transfection, the resulting conditioned medium was collected, filtered, and mixed with polybrene (8 μg/ml). Target cells were incubated in viral-containing supernatants for 48 h, and cells expressing nonsilencing or FAK-specific shRNAs were isolated by puromycin selection (5 μg/ml) for 14 days. Afterward, puromycin-resistant NMuMG and 4T1 cells were assayed for FAK-deficiency by immunoblotting with anti-FAK antibodies, as described later.

### Immunoblot and immunoprecipitation assays

For FAK immunoblots, β_3 _integrin-expressing NMuMG cells were trypsinized, pelleted, and maintained in a nonadherent state for 4 h in serum-reduced media (0.5%). Afterward, the cells either were immediately harvested or were replated in the absence or presence of TGF-β_1 _(5 ng/ml) for an additional 4 h, at which point they were harvested to detect differences in FAK phosphorylation and expression by immunoblotting (see later). Whole-cell extracts prepared from normal and malignant MECs were immunoprecipitated with antibodies against TβR-II, Grb2, and β_3 _integrin (Santa Cruz Biotechnology, Santa Cruz, CA), and the resulting immunocomplexes were immunoblotted various antibodies listed later [19]. Where specified, 4T1 cells were incubated for 18 h in the absence or presence of the FAK inhibitors, PF-562271 or PF-573228 (Pfizer, Inc., New York, NY) at the indicated concentrations before immunoprecipitation of β_3 _integrin. NMuMG cells also were incubated in serum-reduced media (0.5% FBS) supplemented with TGF-β_1 _(5 ng/ml) for 18 h in the absence or presence of the Src inhibitor, PP2 (10 μg/ml; Calbiochem, Temecula, CA). For all cell-signaling experiments, 4T1 or NMuMG cells were serum starved (0 FBS) or deprived (0.5% FBS), respectively, for 6 h before TGF-β_1 _stimulation (5 ng/ml). Control and FAK-deficient 4T1 cells were incubated for up to 48 hours with TGF-β_1 _(5 ng/ml) and detergent-solubilized whole-cell extracts were prepared and immunoblotted for E-cadherin (E-Cad; BD Biosciences, San Jose, CA). Last, 4T1 cells were incubated with TGF-β_1 _(5 ng/ml) for 24 h in serum-free medium, and the resulting conditioned medium was precipitated with 0.01% sodium deoxycholate/6.25% trichloracetic acid and immunoblotted for plasminogen activator inhibitor-1 (PAI-1; Santa Cruz Biotechnology).

Cell extracts were prepared by harvesting NMuMG and 4T1 cells on ice in 3-D RIPA buffer (50 m*M *Tris, 150 m*M *NaCl, 6 m*M *sodium deoxycholate, 1.0% NP-40, 0.1% SDS, pH 7.4) supplemented with protease inhibitor cocktail (Sigma, St. Louis, MO) and phosphatase inhibitors (10 m*M *sodium orthovanadate, 40 m*M *β-glycerophosphate, 20 m*M *NaF), and subsequently were clarified by microcentrifugation before immunoblotting with the following primary antibodies (dilution): (a) anti-phospho-Y397-FAK (1:1000; Cell Signaling, Danvers, MA); (b) anti-phospho-Y577-FAK (1:1000; Cell Signaling); (c) anti-phospho-Y925-FAK (1:1,000; Cell Signaling); (d) anti-phospho-p38 MAPK (1:500; Cell Signaling); (e) anti-phospho-Smad2 (1:1,000; Cell Signaling); (f) anti-phospho-Smad3 (1:500; Cell Signaling); (g) anti-E-cadherin (1:2,500 BD Biosciences); (h) anti-PAI-1 (1:1,000; Santa Cruz Biotechnology); (i) anti-FAK (1:1,000; Santa Cruz Biotechnology); (j) anti-β-actin (1:1,000; Santa Cruz Biotechnology); (k) anti-p38 MAPK (1:1,000; Santa Cruz Biotechnology); (l) anti-Smad2/3 (1:1,000; Cell signaling); (m) anti-phospho-Y416-Src (1:1,000; Cell Signaling); and (n) anti-Src (1:1,000, Cell Signaling).

### Cell migration and invasion assays

Confluent NMuMG cell cultures were wounded with a micropipette tip (200 μl) and immediately placed in 1% serum-containing medium supplemented with or without TGF-β_1 _(5 ng/ml) or the TβR-I inhibitor, SB-431542 (10 μ*M*; Sigma). Bright-field images of wounded monolayers were obtained immediately after wounding (T_0_) and at various times thereafter as indicated. The extent of wound closure was quantified by obtaining three wound measurements for each of three random fields (x100) per wound, and all wound conditions were performed in triplicate. Measurements were taken by using the SlideBook Imaging Software (Intelligent Imaging Innovations, Inc., Denver, CO). The ability of TGF-β_1 _(5 ng/ml) to alter the invasion of 4T1 cells (50,000 cells/well) was analyzed by using a modified Boyden Chamber assay, as described previously [19].

### Luminescent reporter gene assays

Alterations in gene expression regulated by TGF-β were assessed by using a reporter gene assay that monitored changes in luciferase expression driven by the synthetic SBE (Smad-binding element) promoter, as described previously [22]. In brief, NMuMG cells (25,000 to 30,000 cells/well) were allowed to adhere overnight to 24-well plates. The following morning, the cells were transiently transfected by overnight exposure to LT1-liposomes (Mirus, Madison, WI) that contained 300 ng/well of pSBE-luciferase cDNA and 50 ng/well of CMV-β-gal cDNA, which was used to control for differences in transfection efficiency. Afterward, the cells were washed twice with PBS and stimulated overnight with TGF-β_1 _(5 ng/ml) in serum-deprived (0.5% FBS) media. Upon completion of agonist stimulation, firefly luciferase and β-gal activities present in detergent-solubilized cell extracts were determined. In addition, 4T1-luciferase cells that stably expressed firefly luciferase under control of the CMV promoter were cultured into 96-well plates at a density of 10,000 cells/well and subsequently were transiently transfected with an SBE reporter plasmid that drove expression of renilla luciferase. The transfectants were stimulated with TGF-β_1 _as described previously, and subsequently were processed for the determination of renilla and firefly luciferase by using the Dual-Glo Assay System (Promega, Madison, WI).

### Real-time PCR analyses

NMuMG and 4T1 cells were stimulated with TGF-β_1 _(5 ng/ml) for 24 h, and total RNA was isolated by using the RNeasy Plus Kit (Qiagen, Valencia, CA). Afterward, total RNA was reverse transcribed by using the iScript cDNA Synthesis System (BioRad, Hercules, CA), and semiquantitative real-time PCR was conducted by using iQ SYBR Green (BioRad) according to the manufacture's recommendations and as described previously [23]. In all cases, differences in RNA concentration were controlled by normalizing individual gene signals to their corresponding GAPDH RNA signals. The oligonucleotide primer pairs used are provided in Additional data file 1.

### Immunofluorescent analyses

NMuMG cells (25,000 cells/well) were allowed to adhere overnight to glass coverslips in a 24-well plate. Afterward, the cells were washed extensively in PBS and immediately stimulated with TGF-β_1 _(5 ng/ml) in serum-deprived (0.5% FBS) media for 18 h. Upon completion of agonist stimulation, the cells were (a) fixed in 4% paraformaldehyde; (b) permeablized in 0.1% Triton X-100; (c) stained with phospho-Y925 FAK antibodies according to the manufacture's instructions (Cell Signaling); and (d) visualized by using FITC-labeled donkey anti-rabbit secondary antibodies (Jackson ImmunoResearch, West Grove, PA). The actin cytoskeleton was visualized by using TRITC-conjugated phalloidin (0.25 μ*M*; Invitrogen) as described previously [19].

### Tumor growth, bioluminescent imaging, and immunohistochemical analyses

Control or various 4T1 derivatives engineered to express firefly luciferase stably were resuspended in sterile PBS (50 μl) and injected orthotopically into the mammary fat-pads (0.5 to 1.0 × 10^4 ^cells/injection) of 6-week-old female Balb/C mice (Jackson Labs, Bar Harbor, ME). Primary tumor growth and metastasis development was assessed by using digital calipers (Fisher Scientific, Waltham, MA), and by weekly bioluminescent imaging on a Xenogen IVIS-200 (Caliper Life Sciences, Hopkinton, MA). Tumor volumes were calculated by using the following equation:

where "x" is the tumor width and "y" is the tumor length. Finally, serial histologic sections of control and FAK-deficient 4T1 tumors removed after the study were stained with phospho-specific antibodies against p38 MAPK and Smad2 and counterstained with hematoxylin, as previously described [17]. Where indicated, mice were treated daily with PF-562271 (50 mg/kg) or vehicle (5% gelucire 44/14; Gattefosse, Saint Priest Cedex, France) by oral gavage. Histologic sections from these studies were stained with antibodies for the F4/80 macrophage marker (IHC Tech, Aurora, CO), or with phospho-specific antibodies against Y397-FAK, as described previously [17]. All animal studies were performed in accordance with the animal protocol procedures approved by the Institutional Animal Care and Use Committee of University of Colorado.

### Statistical analysis

Statistical values were defined by using an unpaired Student's *t*-test; a *P *value <0.05 was considered significant. *P *values for all experiments analyzed are indicated.

## Results

### TGF-β-stimulated FAK activation and stabilization is dependent on Src and β_3 _integrin

**Figure 1:**
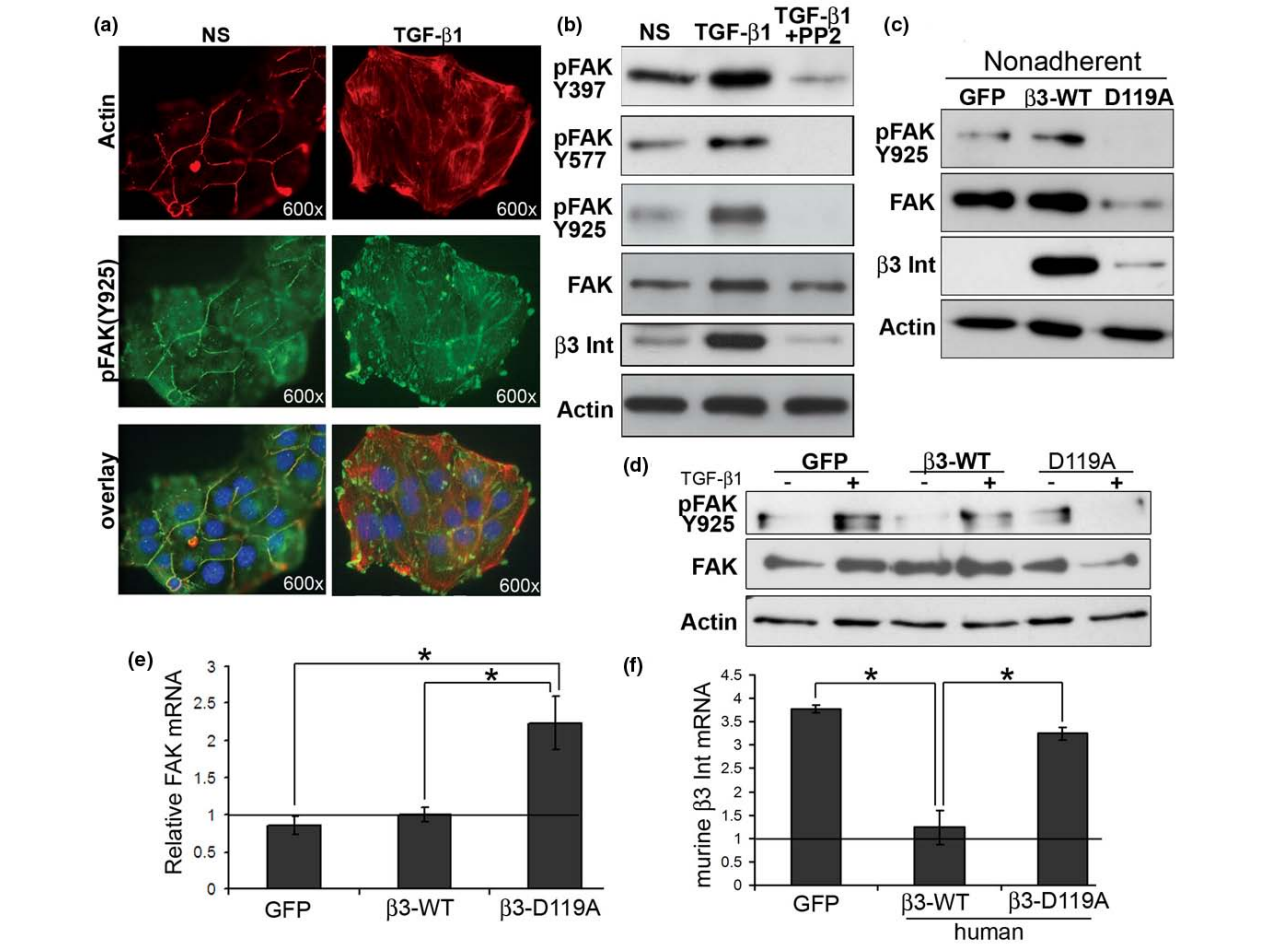
Transforming growth factor (TGF)-β-stimulated focal adhesion kinase (FAK) phosphorylation depends on Src and β_3 _integrin. **(a) **Fluorescently labeled phalloidin (red) and phosphorylated Y925-FAK (green) staining in quiescent (NS) NMuMG cells showed normal cortical actin distribution that colocalized with phosphorylated FAK at cell-cell junctions and borders. Administering TGF-β_1 _(5 ng/ml) for 18 hours promoted F-actin stress fiber formation, and reorganized phosphorylated FAK. DAPI counterstaining, shown in blue (overlay). Data are representative images from a single experiment that was repeated 3 times. **(b) **Immunoblot analysis of cell extracts prepared from NMuMG cells stimulated with TGF-β_1 _as in (a) showed that TGF-β treatment induced the phosphorylation of FAK at positions Y397, Y577, and Y925. Total FAK protein levels also were increased. Administration of the Src inhibitor, PP2 (10 μg/ml) for the duration of the TGF-β_1 _treatment (18 hours), prevented the phosphorylation of FAK at Y577 and Y925, but not at its autophosphorylation site, Y397. In addition, PP2 treatment prevented the induction of β_3 _integrin expression in NMuMG cells stimulated with TGF-β_1_. Differences in protein loading were monitored by reprobing stripped membranes with antibodies against β-actin. Data are representative images from a single experiment that was repeated 3 times. **(c) **FAK phosphorylation and its overall expression was decreased in nonadherent NMuMG cells (4 hours) expressing mutant β_3 _integrin (D119A), but not its wild-type counterpart (β3-WT). Protein loading was monitored by reprobing stripped membranes with antibodies against β-actin. Data are representative images from a single experiment that was repeated 3 times. **(d) **NMuMG cells were treated as in (c) and then replated for 4 hours in the absence or presence of TGF-β_1 _(5 ng/ml) before analyzing FAK phosphorylation and expression. Protein loading was monitored by reprobing stripped membranes with antibodies against β-actin. Data are representative images from a single experiment that was repeated twice with identical results. **(e, f) **Polyclonal populations of control (*i.e*., GFP), WT-β_3 _integrin (β_3_-WT)-, or D119A-β_3 _integrin (β_3_-D119A)-expressing NMuMG cells were stimulated with TGF-β_1 _(5 ng/ml) for 24 hours, and total RNA was isolated and analyzed with semiquantitative real-time polymerase chain reaction (PCR) to monitor FAK (e) and murine β_3 _integrin (f) expression. Data are expressed as the mean (± SEM; n = 3) relative FAK and β_3 _integrin transcript levels measured in response to TGF-β_1_, as compared with their unstimulated counterparts. (*P < 0.05).

NMuMG cells exhibit several distinct morphologic features in response to TGF-β, most notably a dramatic reorganization of the actin cytoskeleton [24]. Figure 1a shows that phosphorylated FAK localized primarily to the periphery in quiescent NMuMG cells, producing a staining pattern very similar to that of F-actin, which was visualized with phalloidin staining (Figure 1a). However, upon TGF-β_1 _stimulation, phosphorylated FAK underwent a dramatic reorganization and localized primarily at the end of actin stress fibers (Figure 1a; overlay). Accordingly, immunoblotting NMuMG cell extracts with a panel of phospho-specific FAK antibodies showed that TGF-β stimulation dramatically increased the phosphorylation of FAK (Figure 1b). Figure 1b also shows that TGF-β stimulation increased FAK protein levels in NMuMG cells and induced an impressive upregulation of β_3 _integrin [19]. Both the increase in FAK phosphorylation and expression, as well as the increase in β_3 _integrin expression were wholly dependent on Src activity, because treating these same cells with the Src inhibitor, PP2, abrogated FAK phosphorylation at the Src-dependent sites (Y577 and Y925) and prevented TGF-β_1_-induced expression of FAK and β_3 _integrin (Figure 1b). Furthermore, in contrast to control (*i.e*., GFP) and WT-β_3 _integrin-expressing NMuMG cells, those engineered to express a signaling-deficient mutant of β_3 _integrin, D119A-β3, exhibited drastically reduced maintenance of FAK protein levels and phosphorylation in response to nonadherent conditions (Figure 1c). To more thoroughly investigate the role of β_3 _integrin in TGF-β-mediated stabilization and phosphorylation of FAK, nonadherent NMuMG cells were replated in the absence or presence of TGF-β_1 _before analyzing FAK expression and phosphorylation by immunoblotting. As shown in Figure 1d, treatment of control (*i.e*., GFP) and WT-β_3 _integrin-expressing NMuMG cells with TGF-β_1 _stimulated increased FAK phosphorylation (Figure 1d). In stark contrast, TGF-β treatment of D119A-β_3 _integrin-expressing NMuMG cells actually decreased their expression and phosphorylation of FAK (Figure 1d). Finally, we conducted real-time PCR for FAK in control (*i.e*., GFP), WT-β_3_, D119A-β_3 _integrin-expressing NMuMG cells. As shown in Figure 1e, chronic TGF-β stimulation (*i.e*., 24 h) had no effect on FAK mRNA levels in control or WT-β_3 _integrin-expressing NMuMG cells; however, these same experimental conditions did increase FAK mRNA expression in D119A-β3 NMuMG cells. These data strongly suggest that (a) upregulated β_3 _integrin expression is required to stabilize FAK protein levels upon TGF-β stimulation, and (b) activated β_3 _integrin signaling acts as a negative-feedback mechanism governing FAK transcription. Along these lines, our use of oligonucleotide sequences that specifically amplified murine β_3 _integrin sequences, not that of recombinant human WT or D119A β_3 _integrin sequences, showed that NMuMG cells engineered to overexpress WT-β_3 _integrin failed to upregulate endogenous murine β_3 _integrin transcripts in response to TGF-β stimulation (Figure 1f). Thus, these findings provide the first evidence that the stability and extent of FAK phosphorylation induced by TGF-β is critically dependent on its ability to upregulate functional β_3 _integrin, and that both of these events require the activity of Src kinase. These data also suggest that FAK may play a critical function with β_3 _integrin and Src [19] in facilitating TGF-β signaling and function in MECs.

### FAK is critically involved in TGF-β-induced p38 MAPK activation and mammary epithelial cell migration

**Figure 2:**
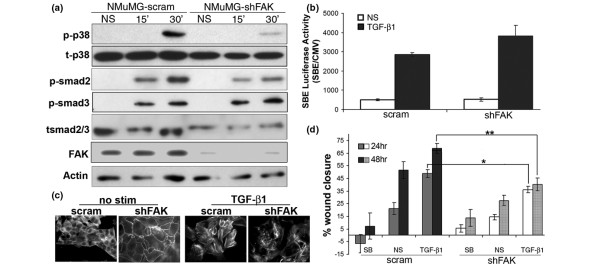
Focal adhesion kinase (FAK) is critically involved in normal mammary epithelial cell (MEC) migration and p38 MAPK signaling stimulated by transforming growth factor (TGF)-β. **(a) **Control (*i.e*., scram) and FAK-deficient (shFAK) NMuMG cells were stimulated with TGF-β_1 _(5 ng/ml) for varying times as indicated, at which point whole-cell extracts were prepared and immunoblotted with phospho-specific antibodies against Smad2 (p-smad2), Smad3 (p-smad3), or p38 MAPK (p-p38) as shown. Immunoblot analysis of total p38 MAPK, Smad2/3, FAK, and β-actin serves as loading controls. Data are representative images from a single experiment that was repeated 3 times. **(b) **Control (*i.e*., scram) and FAK-deficient (shFAK) NMuMG cells were transiently transfected with pSBE-luciferase and pCMV-β-gal cDNAs, and subsequently were stimulated with TGF-β_1 _(5 ng/ml) for 18 hours. Afterward, luciferase and β-gal activities present in detergent-solubilized cell extracts were measured. Data are expressed as the mean (± SEM) ratios of luciferase:β-gal activity observed in three independent experiments completed in triplicate. **(c) **Fluorescently labeled phalloidin staining showed the formation of F-actin stress fibers in control (*i.e*., scram) NMuMG cells treated for 18 hours with TGF-β_1 _(5 ng/ml). FAK-deficiency (shFAK) significantly impaired the ability of TGF-β_1 _to induce stress fibers, as well as disrupted normal actin cytoskeletal architecture. Data are representative images from a single experiment that was repeated twice. **(d) **Control (*i.e*., scram) and FAK-deficient (shFAK) NMuMG cell migration into denuded wounds over a 48-hour period in the absence (NS) or presence of either the TβR-I inhibitor, SB-431542 (10 μ*M*, SB) or TGF-β_1 _(5 ng/ml). Data depict the mean (± SEM) percentage of wound closure measured in five independent experiments. FAK deficiency inhibited tonic and TGF-β_1_-stimulated NMuMG cell migration. In addition, tonic NMuMG cell migration was inhibited by SB-431542, indicating disruption of autocrine TGF-β signaling in wounded cultures (*P < 0.05; **P < 0.005).

To assess the role of FAK in mediating downstream TGF-β signaling events, we next used shRNAs to deplete stably the expression of FAK in NMuMG cells (Figure 2a). As shown in Figure 2a and 2b, FAK-deficiency had no effect on canonical Smad2/3 activity stimulated by TGF-β, but did markedly disrupt the coupling of TGF-β to the noncanonical p38 MAPK pathway. Furthermore, the quiescent architecture of the actin cytoskeleton, as well as TGF-β-induced actin stress fibers were severely disrupted upon FAK depletion (Figure 2c). We also examined the impact of FAK deficiency on the ability of TGF-β to stimulate MEC migration. To do so, confluent monolayers of control or FAK-deficient NMuMG cells were wounded with a micropipette tip, and the extent of MEC migration into the denuded area was measured at various times thereafter. Stimulating FAK-deficient NMuMG cells with TGF-β_1 _enhanced their wound-healing response [25], although at a significantly reduced rate as compared with control (*i.e*., scrambled shRNA) NMuMG cells (Figure 2d), suggesting that FAK plays a critical role in TGF-β-induced MEC migration. Accordingly, administration of the TβR-I inhibitor, SB-431542, inhibited control NMuMG cell wound closure, thereby identifying a role for autocrine TGF-β signaling in mediating the closure of MEC wounds. Interestingly, FAK-deficient NMuMG cells were refractory to administration of the TβR-I inhibitor (Figure 2d), suggesting that these cells have adapted a less-efficient mechanism of migration that is no longer dependent on the activities of TGF-β and FAK. Finally, as wound closure is driven by both cell migration and proliferation, the decreased growth rate of FAK-deficient cells (see Additional data file 2a) may contribute to their reduced wound-healing response. However, this does not appear to be the case in NMuMG cells, as control and FAK-deficient cells exhibit similar cytostatic responses to high-dose TGF-β1 treatment (5 ng/ml; see Additional data file 2b), which indicates that the difference in wound healing between control and FAK-deficient cells reflects alterations in their ability to migrate, not to proliferate (Figure 2d). Taken together, these data strongly suggest that FAK is directly involved in mediating TGF-β-induced MEC migration.

### FAK is required for oncogenic signaling by TGF-β

**Figure 3:**
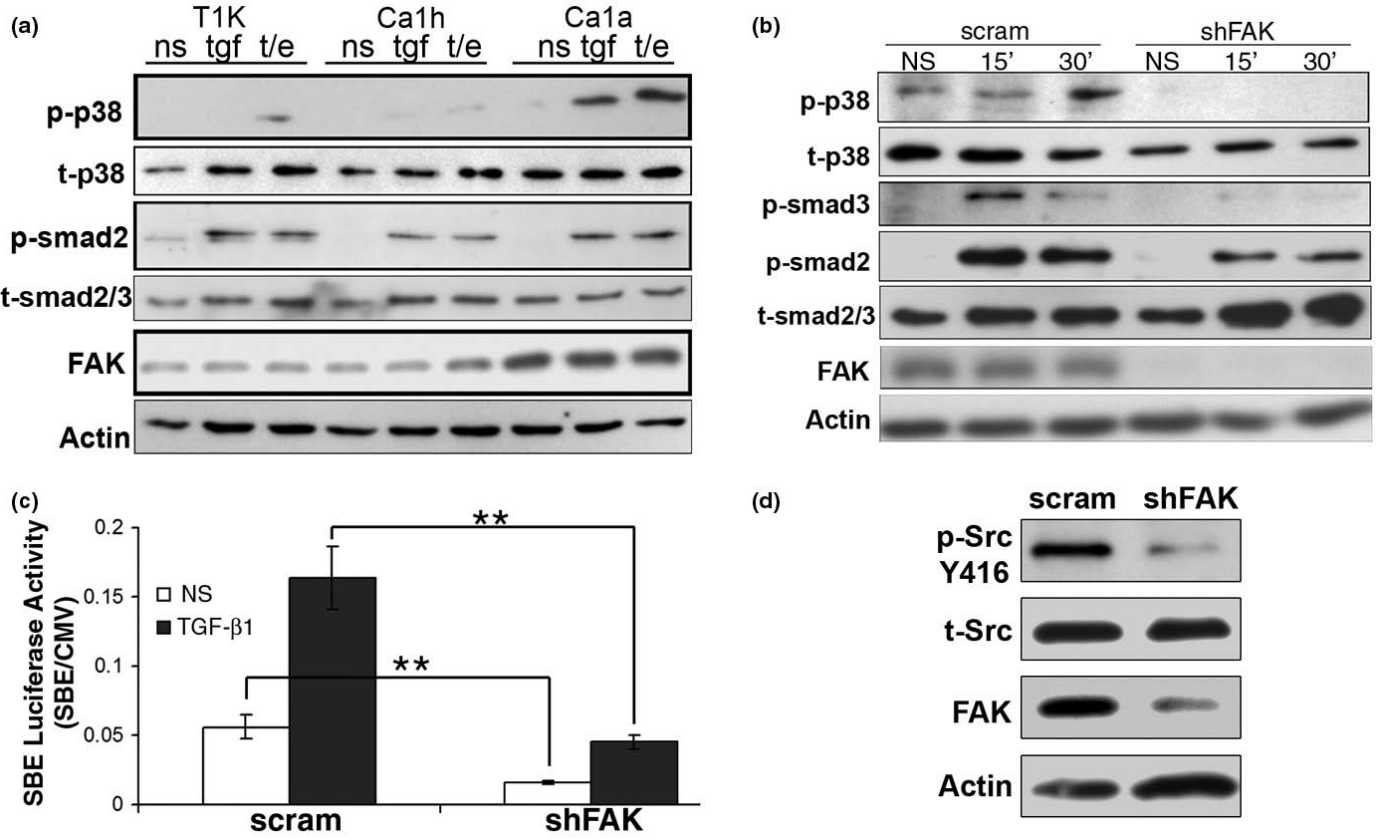
Focal adhesion kinase (FAK) is essential in mediating oncogenic signaling by transforming growth factor (TGF)-β. **(a) **Increased FAK expression correlates with TGF-β_1_-mediated activation of p38 MAPK in metastatic human breast cancer cells. The human MCF10A mammary carcinoma progression system (T1k, well differentiated; Ca1h, premalignant; and Ca1a, metastatic) was stimulated with TGF-β_1 _(5 ng/ml; tgf), or with TGF-β_1 _(5 ng/ml) in combination with epidermal growth factor (50 ng/ml; t/e) for 30 minutes and subsequently analyzed for phosphorylation of p38 MAPK (p-p38) or Smad2 (p-smad2), as indicated. Increased FAK expression was noted in metastatic Ca1a cells. Protein loading was monitored by reprobing stripped membranes with antibodies against total p38 MAPK (t-p38), total Smad2/3 (t-smad2/3), and β-actin. Data are representative images from a single experiment that was repeated twice. **(b) **Control (*i.e*., scram) and FAK-deficient (shFAK) 4T1 cells were stimulated with TGF-β_1 _(5 ng/ml) for varying times, as indicated. Afterward, cell extracts were prepared and immunoblotted with phospho-specific antibodies against Smad2 (p-smad2), Smad3 (p-smad3), or p38 MAPK (p-P38), as shown. Reprobing stripped membranes with antibodies against total p38 MAPK (t-p38), total Smad2/3 (t-smad2/3) FAK, and β-actin served as loading controls. Data are representative images from a single experiment that was repeated 3 times. **(c) **Control (*i.e*., scram) and FAK-deficient (shFAK) 4T1-luciferase cells were transfected with pSBE-renilla luciferase and stimulated with TGF-β_1 _(5 ng/ml) before measuring firefly and renilla luciferase activities. Data are expressed as the mean (± SEM) ratios of renilla:firefly luciferase activity observed in three independent experiments completed in triplicate. (*P < 0.05; **P < 0.0001). **(d) **Src phosphorylation (p-Src-Y416) was decreased in FAK-depleted (shFAK) 4T1 cells as compared with their control (*i.e*., scram) counterparts. Immunoblots were stripped and reprobed with antibodies against Src (t-Src), FAK, and β-actin as loading controls. Data are representative images from a single experiment that was repeated 3 times.

Imbalances between canonical and noncanonical TGF-β signaling contribute to mammary tumorigenesis [4]. We illustrated this shift in TGF-β signaling by using the human MCF10A breast cancer progression model that consists of indolent (T1K), malignant nonmetastatic (Ca1h), and malignant metastatic (Ca1a) cells [26,27]. With this model system, we observed an enhanced ability of TGF-β to activate specifically p38 MAPK, but not Smad2, in a manner correlating with increasing metastatic potential (Figure 3a). Importantly, the improved coupling of TGF-β to p38 MAPK activation in malignant metastatic Ca1a cells correlated with a marked upregulation of FAK expression as compared with their premetastatic counterparts (Figure 3a). These findings are consistent with the notion that the acquisition of metastasis by breast cancer cells coincides with their elevated expression of FAK, which enhances p38 MAPK activation by TGF-β and its pro-metastatic activities.

To investigate the merits of this supposition, we compared the ability of control and FAK-deficient metastatic breast cancer cells to activate Smad2/3 and p38 MAPK in response to TGF-β. We found that FAK deficiency dramatically not only decreased basal p38 MAPK phosphorylation, but also completely abrogated the ability of TGF-β to activate p38 MAPK (Figure 3b). Interestingly, in contrast to what we observed in NMuMG cells (Figure 2a and 2b), the coupling of TGF-β to both Smad2 and Smad3 were also decreased in FAK-deficient 4T1 cells (Figures 3b and 3c). These data stress the increased dependence of metastatic breast cancer cells on FAK to facilitate not only noncanonical (p38 MAPK), but also canonical (Smad2/3) TGF-β signaling (Figure 3).

**Figure 4:**
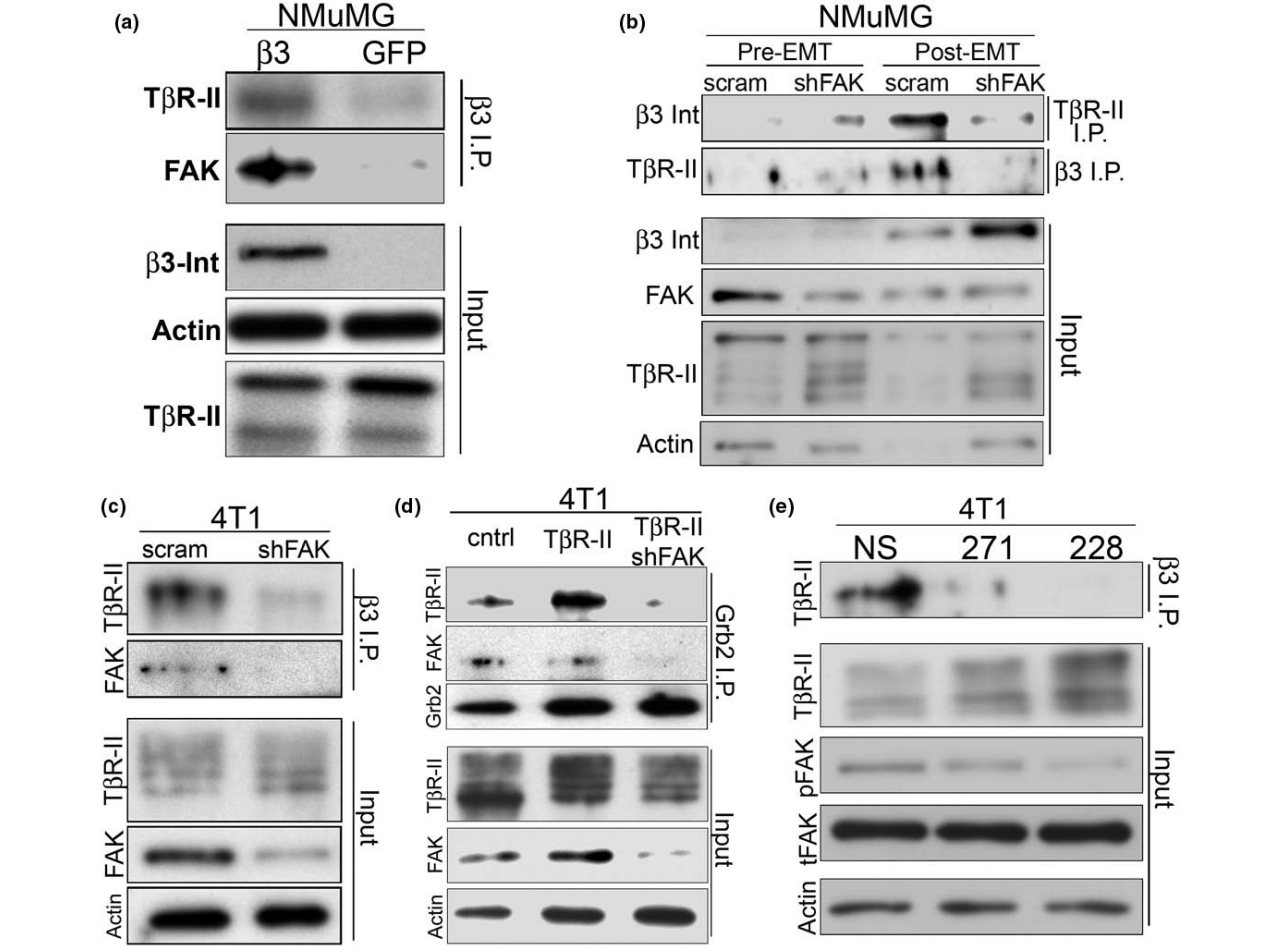
Focal adhesion kinase (FAK) activity coordinates the formation of β_3 _integrin:TβR-II complexes. **(a) **β_3 _integrin was immunoprecipitated (β_3 _I.P.) from control (*i.e*., GFP) or β_3 _integrin-expressing NMuMG cell extracts, and the resulting immunocomplexes were immunoblotted with antibodies against TβR-II and FAK, as indicated. Direct immunoblot analysis of an aliquot of the prepared cell extracts (Input) served to monitor the total levels of TβR-II, β_3 _integrin, and β-actin. Data are representative images from a single experiment that was repeated 3 times and show that FAK was present in β_3 _integrin:TβR-II complexes. **(b) **β_3 _integrin (β_3 _I.P.) or TβR-II (TβR-II I.P.) were immunoprecipitated from control (*i.e*., scram) and FAK-deficient (shFAK) NMuMG cell extracts before (pre-EMT) or after (post-EMT) their induction of EMT by TGF-β_1 _(5 ng/ml, 24 hours), and the resulting immunocomplexes were reciprocally immunoblotted for β_3 _integrin or TβR-II. Direct immunoblot analysis of an aliquot of the prepared cell extracts (Input) served to monitor the total levels of TβR-II, β_3 _integrin, FAK, and β-actin. **(c) **β_3 _integrin was immunoprecipitated (β_3 _I.P.) from control (scram) and FAK-deficient (shFAK) 4T1 cell extracts, and the resulting immunocomplexes were immunoblotted for TβR-II and FAK, as indicated. Direct immunoblot analysis of an aliquot of the prepared cell extracts (Input) served to monitor the total levels of FAK, TβR-II, and β-actin. Data are representative images from a single experiment that was repeated 4 times and show that FAK deficiency inhibits the formation of β_3 _integrin:TβR-II complexes. **(d) **Grb2 was immunoprecipitated (Grb2 I.P.) from vector control (cntrl), TβR-II- (TβR-II), and FAK-deficient and TβR-II-expressing (TβR-II/shFAK) 4T1 cell extracts, and the resulting immunocomplexes were immunoblotted for TβR-II, FAK, and Grb2, as indicated. Direct immunoblot analysis of an aliquot of the prepared cell extracts (Input) served to monitor the total levels of TβR-II, FAK, and β-actin. Data are representative images from a single experiment that was repeated 3 times and show that FAK deficiency inhibits the interaction between Grb2 and TβR-II. **(e) **β_3 _integrin was immunoprecipitated (β_3 _I.P.) from unstimulated (NS) cell extracts derived from 4T1 cells and cells incubated for 18 hours with the FAK inhibitors PF-562271 (271) or PF-573228 (228), as indicated. The resulting immunocomplexes were immunoblotted for TβR-II. Direct immunoblot analysis of an aliquot of the prepared cell extracts (Input) served to monitor the levels of FAK phosphorylated at Y397 (pFAK), total FAK (tFAK), TβR-II, and β-actin. Data are representative images from a single experiment that was repeated 3 times and show that FAK PTK activity is required for the formation of β_3 _integrin:TβR-II complexes.

Previously, we established Src as being essential for the ability of TGF-β to stimulate p38 MAPK in MECs [19]. To investigate the role of FAK in this mechanism, we now analyzed the phosphorylation and activation status of Src (*i.e*., phosphorylation at Y416) in control and FAK-depleted cells, which showed that FAK deficiency rendered Src hypophosphorylated in 4T1 cells (Figure 4d). Collectively, these findings are the first to demonstrate the increasing dependence of metastatic breast cancer cells on the reciprocal activation between FAK and Src in mediating downstream TGF-β signaling [28].

### FAK expression and kinase activity are required for the aberrant formation of β_3 _integrin:TβR-II signaling complexes

We next sought to address the mechanism by which FAK facilitates oncogenic TGF-β signaling. Previously, we observed β_3 _integrin to interact aberrantly with TβR-II [19], resulting in the promotion of MAPK signaling by TGF-β [17,18]. Indeed, robust quantities of FAK were detected in β_3 _integrin:TβR-II complexes specifically in NMuMG cells engineered to overexpress β_3 _integrin (Figure 4a; [19]). Furthermore, the formation of β_3 _integrin:TβR-II complexes was readily induced in NMuMG cells subsequent to their induction of EMT by TGF-β (Figure 4b; [19]). However, this same TGF-β treatment protocol failed to induce β_3 _integrin:TβR-II interaction in FAK-depleted NMuMG cells, as (a) β_3 _integrin immunocomplexes isolated from FAK-deficient NMuMG cells no longer included TβR-II, and (b) TβR-II immunocomplexes no longer included β_3 _integrin (Figure 4b). Moreover, whereas the formation of β_3 _integrin:TβR-II complexes was dependent on EMT in NMuMG cells, these same complexes were observed to form constitutively in 4T1 cells (Figure 4c). Importantly, depleting FAK expression in 4T1 cells also reduced the interaction between β_3 _integrin and TβR-II, as β_3 _integrin immunocomplexes isolated from FAK-deficient 4T1 cells no longer included TβR-II (Figure 4c). The formation of β_3 _integrin:TβR-II complexes leads to Src-mediated phosphorylation of TβR-II on Y284, which coordinates the recruitment and binding of Grb2 to TβR-II [18]. We now show that FAK deficiency prevented the interaction between TβR-II and Grb2, as Grb2 immunocomplexes isolated from FAK-depleted 4T1 cells no longer included TβR-II (Figure 4d).

Finally, we found that the PTK activity of FAK was absolutely required for the formation of β_3 _integrin:TβR-II complexes, as treatment of 4T1 cells with an effective concentration (see Additional data file 3d) of FAK inhibitors (PF-562271 and PF-573228) similarly abolished the interaction between β_3 _integrin and TβR-II (Figure 4e). Taken together, these findings demonstrate that FAK expression and activity are required for the formation of β_3 _integrin:TβR-II:Grb2 complexes (Figure 4) and, consequently, for the initiation of aberrant oncogenic TGF-β signaling (Figure 3).

### FAK is critically involved in TGF-β-stimulated invasion of malignant MECs

**Figure 5:**
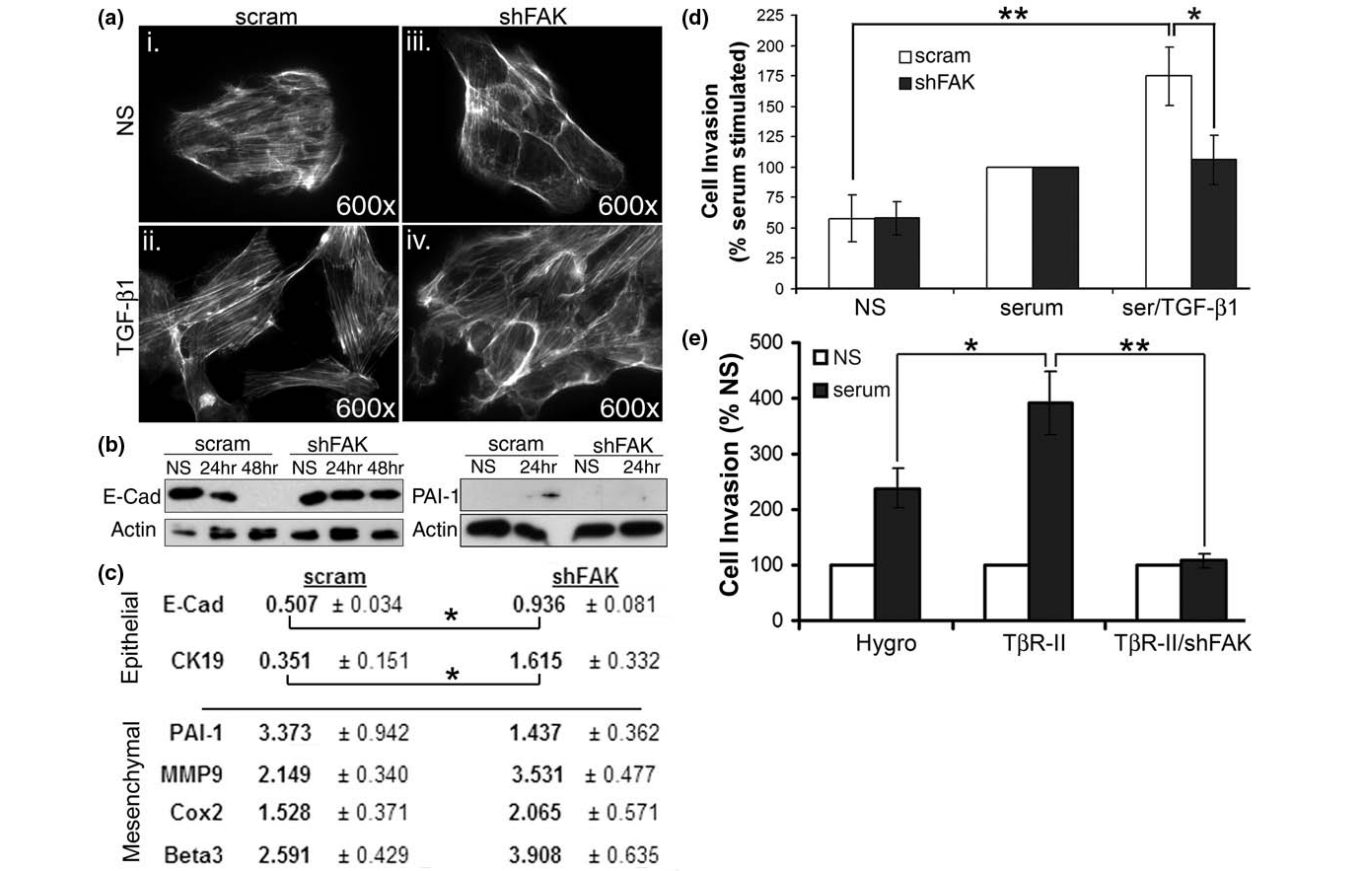
Focal adhesion kinase (FAK) is critical for TGF-β stimulation of invasion in malignant mammary epithelial cells (MECs). **(a) **Fluorescent-conjugated phalloidin staining of control (*i.e*., scram) and FAK-deficient (shFAK) 4T1 cells indicated that FAK deficiency (shFAK) preserved cortical actin staining at cell-cell borders with TGF-β_1 _(5 ng/ml) stimulation. Data are representative images from a single experiment that was repeated twice. **(b) **Control (*i.e*., scram) and FAK-deficient (shFAK) 4T1 cells were stimulated with TGF-β_1 _(5 ng/ml) for varying times, as indicated. Afterward, cell extracts or supernatants were immunoblotted with antibodies against E-cadherin (E-cad) or plasminogen activator inhibitor-1 (PAI-1), respectively. Immunoblotting for β-actin served as a loading control. Data are representative images from a single experiment that was repeated twice and show that FAK deficiency inhibited epithelial-mesenchymal transition (EMT) stimulated by TGF-β. **(c) **Control (*i.e*., scram) and FAK-depleted (shFAK) 4T1 cells were stimulated with TGF-β_1 _(5 ng/ml) for 24 hours. Afterward, the downregulation of the epithelial markers E-Cad and cytokeratin-19 (CK19), and the upregulation of the mesenchymal markers, PAI-1, matrix metalloproteinase 9 (MMP9), cyclooxygenase-2 (Cox2), and β_3 _integrin (Beta3) were analyzed with semiquantitative real-time PCR. Data are expressed as the mean fold change (± SEM) in gene expression relative to unstimulated cells observed in at least three independent experiments (*P < 0.05). **(d) **FAK deficiency specifically inhibited cell invasion induced by TGF-β. Data are the mean (± SEM) invasion of each 4T1 cell line normalized to serum-stimulated invasion observed in three independent experiments completed in triplicate. (*P < 0.05; **P < 0.005). **(e) **FAK deficiency reversed TβR-II-mediated cellular invasion. Data are expressed as the mean (± SEM) invasion of each 4T1 cell line relative to its unstimulated counterpart (NS) observed in three independent experiments completed in triplicate. (*P < 0.05; **P < 0.001).

To elucidate the significance of FAK in mediating oncogenic TGF-β signaling, we first compared the ability of TGF-β to induce EMT in control and FAK-deficient 4T1 cells. Figure 5a shows that FAK-depleted 4T1 cells failed to undergo the characteristic cell scattering that is normally associated with the induction of EMT (Figure 5a). In contrast to control cells, FAK-depleted 4T1 cells maintained cell-cell junctions that, in many respects, reflect the cortical actin patterns observed in unstimulated normal MECs (Figure 1a). We corroborated these morphologic findings by examining the differential expression of several genes associated with EMT in control and FAK-deficient 4T1 cells before and after their treatment with TGF-β. In doing so, we observed TGF-β administration to reduce dramatically the 4T1 cell expression of E-cadherin (E-cad) protein and to increase the amount of PAI-1 protein in the conditioned media (Figure 5b). Importantly, both TGF-β-dependent responses were lost in 4T1 cells depleted for FAK expression (Figure 5b).

More thoroughly to characterize the role of FAK in TGF-β1-induced EMT, we also examined a panel of EMT markers by real-time PCR. As shown in Figure 5c, the downregulation of the epithelial markers E-cad and cytokeratin-19, which are characteristic features of EMT induced by TGF-β, was effectively prevented by depletion of FAK. Most interestingly, with the exception of PAI-1, the upregulation of mesenchymal markers was not appreciably affected by FAK deficiency (Figure 5c). The maintenance of an epithelial gene-expression profile is consistent with the epithelial morphology of FAK-depleted 4T1 cells (Figure 5a), and is further supported by a recent study in hepatocytes showing that the expression of a dominant-negative FAK prevents the downregulation of epithelial gene expression without affecting the ability of TGF-β to induce mesenchymal gene expression [29].

Consistent with the switch of TGF-β from a tumor suppressor to a tumor promoter, we and others observed TGF-β to induce the invasion of breast cancer cells, a result that is not recapitulated by normal MECs [18,30]. We therefore monitored the ability of control and FAK-deficient 4T1 cells to invade synthetic basement membranes in response to TGF-β. Figure 5d shows that whereas FAK-deficiency failed to affect the invasion of 4T1 cells induced by a nonspecific serum stimulus, this same cellular condition abrogated the ability of 4T1 cells to undergo enhanced invasion in response to TGF-β (Figure 5d). Previous findings by our laboratory established a model whereby constitutive expression of TβR-II increases the invasion of 4T1 cells [18]. Importantly, depletion of FAK in "hypermetastatic" 4T1-TβR-II cells actually reversed the affects of TβR-II expression, as TβR-II-shFAK cells completely failed to invade to a serum stimulus (Figure 5e). Taken together, these data identify FAK as an essential player in mediating carcinoma cell EMT and invasion induced specifically by TGF-β.

### FAK inhibition reduces breast cancer growth and metastasis

**Figure 6:**
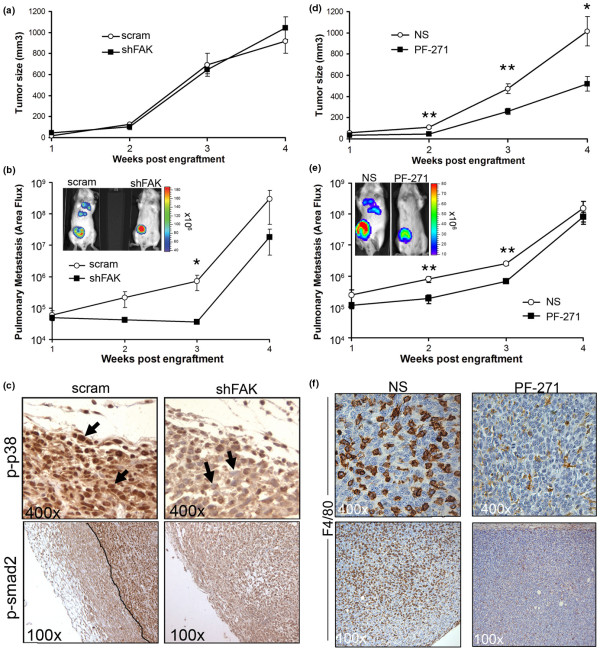
Focal adhesion kinase (FAK) mediates tumor cell autonomous-driven metastasis and systemically driven mammary tumor growth. **(a) **Control (*i.e*., scram) and FAK-deficient (shFAK) 4T1 cells were engrafted onto the mammary fat pads of 6-week-old Balb/C mice. Data are expressed as the mean (± SEM) tumor volumes (n = 5) measured at the indicated time points after engraftment. **(b) **FAK-deficient 4T1 tumors displayed significantly decreased pulmonary metastasis. Data are the mean (± SEM) pulmonary area flux units detected in Balb/C mice (n = 5) imaged at the indicated time points after engraftment (*P < 0.05). Shown are representative mice engrafted with control (*i.e*., scram) and FAK-deficient (shFAK) 4T1 cells (inset). **(c) **Immunostaining primary tumor sections with phospho-specific antibodies against p38 MAPK (p-p38) or Smad2 (p-smad2) showed intense nuclear localization of phosphorylated p38 MAPK at the invasive front of control (*i.e*., scram) tumors (black arrows). Tumors derived from FAK-deficient 4T1 cells exhibit significantly reduced phosphorylation and nuclear accumulation of p38 MAPK (black arrows). Similarly, abundant phosphorylation of Smad2 (p-smad2) was readily detected near the invasive front of control (*i.e*., scram) 4T1 tumors (right of the line), but not in tumors arising from their FAK-deficient (shFAK) counterparts. **(d) **4T1 cells were engrafted onto the mammary fat pads of 6-week-old Balb/C mice. One week after engraftment, the mice were left untreated (NS) or treated with the FAK inhibitor PF-562271 (PF-271; 50 mg/kg/day) for the duration of the experiment. Data are expressed as the mean (± SEM) tumor volumes (n = 5) measured at the indicated time points after engraftment (*P < 0.05; **P < 0.005). **(e) **Mice treated with PF-562271 as in panel (d) displayed significantly decreased pulmonary metastasis. Data are expressed as the mean (± SEM) pulmonary area flux units detected in Balb/C mice (n = 5) imaged at the indicated time points after engraftment (**P < 0.005). Shown are representative untreated (NS) or PF-562271 (PF-271)-treated mice (inset). **(f) **Control (NS) 4T1 mammary fat-pad tumors displayed a high degree of macrophage infiltration, as demonstrated by immunostaining for the F4/80 antigen. Macrophage infiltration was greatly reduced in tumor-bearing mice treated with PF-562271 (PF-271).

Recent data suggest that FAK is required for mammary tumor progression and metastasis [10,11,15]; however, the precise mechanisms whereby FAK promotes tumor progression remain to be elucidated. Although FAK depletion had no affect on primary tumor growth (Figure 6a), bioluminescent imaging of mice bearing 4T1 tumors did show that pulmonary metastasis was reduced significantly upon FAK depletion (Figure 6b). In accordance with our *in vitro *findings (Figure 3), immunohistochemistry of 4T1 tumors demonstrated a dramatic decrease in the phosphorylation of p38 MAPK and Smad2 with FAK depletion (Figure 6c). Thus, these findings suggest that FAK plays a critical role in regulating TGF-β signaling and the metastasis of mammary tumors in mice.

In contrast to tumor cell depletion of FAK, therapeutic administration of the FAK inhibitor, PF-562271, significantly decreased the growth of primary 4T1 tumors (Figure 6d). The reduction in 4T1 tumor growth likely reflects diminished PTK activity of FAK, as tumors from biopsies of mice treated with PF-562271 possessed significantly less phosphorylated FAK as compared with their vehicle-treated counterparts (see Additional data file 3e). Moreover, PF-562271 decreased pulmonary metastasis in a fashion highly reminiscent of that observed in tumors depleted in FAK expression (Figure 6e). The difference in primary tumor growth between FAK-depleted cells (Figure 6a) and systemic FAK inhibition by PF-562271 (Figure 6d) suggests that FAK plays an important role in governing the composition or activity or both of nontumor cells in the tumor microenvironment, including the potential recruitment of systemic cell populations required for optimal mammary tumor growth and progression. Accordingly, we observed 4T1 tumors to exhibit strong staining for the macrophage marker F4/80, a result that was not recapitulated with PF-562271 administration (Figure 6f). Thus, we show for the first time that, in addition to the critical roles FAK plays in directing carcinoma cell function and behavior, the PTK activity of FAK is also clearly required for regulating innate immunity within the microenvironments of developing and progressing mammary tumors.

**Figure 7:**
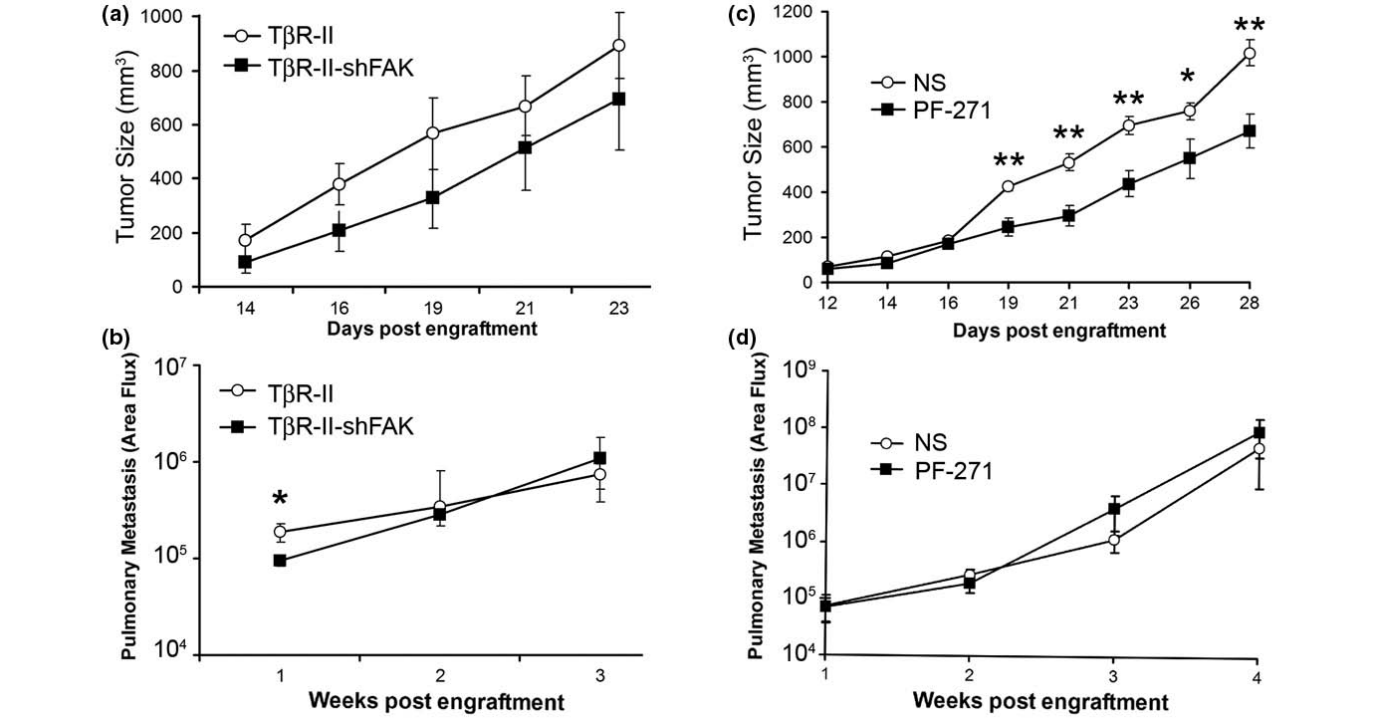
TβR-II-dependent, early primary tumor cell dissemination is dependent on focal adhesion kinase (FAK). **(a) **TβR-II (TβR-II)- or TβR-II-expressing/FAK deficient (TβR-II-shFAK) 4T1 cells were engrafted onto the mammary fat pads of 6-week-old Balb/C mice. Data are expressed as the mean (± SEM) tumor volumes (n = 5) measured at the indicated time points after engraftment. **(b) **FAK-deficient TβR-II tumors displayed significantly decreased primary tumor dissemination 1 week after tumor cell engraftment. Data are expressed as the mean (± SEM) pulmonary area flux units detected in Balb/C mice (n = 5) imaged at the indicated time points after engraftment (*P < 0.05). **(c) **One week after engraftment, 4T1-TβR-II tumor-bearing mice were left untreated (NS) or treated with the FAK inhibitor PF-562271 (PF-271; 50 mg/kg/day) for the duration of the experiment, as described in Figure 6c and 6d. Data are expressed as the mean (± SEM) tumor volumes (n = 5) measured at the indicated time points after engraftment (*P < 0.05; **P < 0.005). **(d) **Mice bearing 4T1-TβR-II tumors that were treated with PF-562271, as in panel (c) (n = 5) displayed similar pulmonary metastasis compared with controls. Data are expressed as the mean (± SEM) pulmonary area flux units detected in Balb/C mice (n = 5) imaged at the indicated time points after engraftment.

We next used the 4T1-TβR-II model to access the specific role of FAK in TGF-β-driven breast cancer metastasis. As shown in Figure 7a, FAK depletion had no effect on primary tumor growth of 4T1-TβR-II cells. Furthermore, although FAK-deficient 4T1-TβR-II cells were still highly metastatic, FAK depletion did significantly decrease the immediate pulmonary dissemination of 4T1-TβR-II cells (Figure 7b). These data suggest that FAK selectively regulates the initial steps of tumor cell dissemination stimulated by TGF-β, a result that is consistent with our findings of the requirement of FAK in (a) mediating EMT stimulated by TGF-β (Figure 5) and (b) preventing primary colonization of breast cancer cells in the lung (Figure 6b, weeks 2 to 3), but not their secondary outgrowth (Figure 6b, weeks 3 to 4). In addition, we found no differences in the ability of control or FAK-deficient 4T1 cells to colonize the lungs after their injection into the tail vein of Balb/C mice (see Additional data file 4c). Taken together, these data suggest that the coupling of TGF-β to FAK promotes the initial invasion and exit of breast cancer cells from the primary tumor site. Moreover, and similar to control 4T1 cells, PF-562271 administration beginning 1 week after engraftment of 4T1-TβR-II cells significantly reduced their growth in mice (Figure 7c); however, this same treatment protocol had no effect on the subsequent metastasis of 4T1-TβR-II cells (Figure 7d).

Collectively, these findings provide the first evidence that FAK activity can be inhibited chemotherapeutically as an effective two-pronged approach to reduce the growth and metastasis of breast cancers. Moreover, these results also show that amplified TGF-β signaling in breast cancer cells is capable of driving early tumor cell dissemination from the primary mammary tumor.

## Discussion

TGF-β is a principal player involved in suppressing mammary tumorigenesis by maintaining the composition of normal MEC microenvironments through its ability to inhibit the proliferation and survival of normal MECs [31,32]. In stark contrast, mammary tumorigenesis has evolved a variety of mechanisms capable of subverting the tumor-suppressing functions of TGF-β and of conferring oncogenic and metastatic properties on this multifunctional cytokine [30,33]. Along these lines, elevated FAK expression is observed in a variety of human cancers, including those of the lung [34], uterus [35], mouth [36], thyroid [37], colon [38], ovary [39], and, most notably, the breast [38,40,41]. Thus, upregulated expression of FAK is associated with the development and progression of human cancers. Accordingly, numerous models have shown that rendering breast cancer cells deficient in FAK inhibits their progression and the acquisition of metastatic phenotypes [10,14,15]. The data shown herein identify FAK as an essential member of oncogenic β_3 _integrin:TβR-II signaling complexes. FAK-deficiency not only prevented the physical interaction between β_3 _integrin and TβR-II, but also abrogated oncogenic signaling by TGF-β and its ability to induce EMT, invasion, and systemic dissemination of breast cancer cells. Thus, FAK is a critical effector of metastasis stimulated by TGF-β in developing and progressing mammary tumors.

Recent data also suggest that FAK mediates *in vitro *TGF-β signaling and gene expression in fibroblasts [42], hepatocytes [29], and mesangial cells [43], further highlighting the biologic importance of this signaling and scaffolding molecule.

Through the use of the recently developed small-molecule inhibitors of FAK (*i.e*., PF-562271 and PF-573228), we specifically defined the PTK activity of FAK as being essential for mediating the formation of β_3 _integrin:TβR-II complexes. Moreover, therapeutic administration of PF-562271 reduced pulmonary metastasis in a manner reminiscent of that observed with total FAK depletion, suggesting that the PTK activity of FAK, as opposed to its scaffolding function, is the major aspect of the this molecule required for cellular metastasis. A clinically relevant finding of our study was that FAK clearly is required for the initiation of TGF-β signaling and its stimulation of EMT and invasion. More important, we showed for the first time that amplified TGF-β signaling through increased TβR-II expression [18] was sufficient in subverting the metastatic benefit of FAK chemotherapies, by using the same treatment protocol that was sufficient in reducing the metastasis of wild-type breast cancer cells. These data suggest that TGF-β drives cellular dissemination from the primary tumor and early metastatic lesion formation, processes that absolutely require FAK expression and PTK activity. This conclusion is wholly supported by recent independent studies showing that both FAK and TGF-β signaling are critically involved in these early steps of tumor dissemination, but not metastatic outgrowth [44,45].

Mechanistically, we showed that FAK becomes activated with TGF-β-mediated induction of EMT, a process that is dependent on Src and β3 integrin (Figure 1[46]). Moreover, we present data to suggest that TGF-β-stimulated upregulation of β_3 _integrin acts as a negative-feedback mechanism regulating the transcription not only of itself, but also that of FAK. Thus, these and our previous findings indicate that the transactivation of FAK and Src facilitates the interaction between β_3 _integrin and TβR-II, leading to phosphorylation of TβR-II at Y284 [18] and its interaction with Grb-2 [17]. Indeed, the formation of integrin:TβR-II complexes, as well as other signaling modules involving TGF-β receptors [47,48], appears to be governed by a variety of protein-protein interactions and posttranslational modifications.

Overall, the formation of these aberrant complexes function to promote the oncogenic activities of TGF-β in developing and progressing breast cancers. Our findings also point to the importance of thoroughly defining the composition and function of these TGF-β signaling complexes in normal and metastatic cells. As such, we show here that β_3 _integrin:TβR-II complexes are present constitutively in metastatic MECs, but only form in normal MECs upon their induction of EMT. Accordingly, disruption of FAK decreases TGF-β-induced Smad2/3 activation and completely abrogates p38 MAPK stimulation in metastatic MECs, whereas FAK depletion in normal MECs only partially blocks TGF-β-induced p38 MAPK activation with no affect on Smad2/3 activity.

Clearly, these data demonstrate the increased dependence of metastatic breast cancer cells on FAK to facilitate oncogenic TGF-β signaling. Moreover, they suggest that targeting FAK and other constituents of the focal adhesion complex, such as integrins, p130Cas, talin, or paxillin, holds the potential to inactivate specifically the oncogenic activities of TGF-β in malignant MECs. Moreover, our findings suggest that the development and use of such a chemotherapeutic regimen would have little impact on altering the tumor-suppressor function of TGF-β in normal MECs.

A scientifically and medically important finding of this study was the difference noted between tumor cell depletion of FAK and systemic FAK inhibition by using PF-562271. We demonstrated a drastic diminution in primary tumor growth in control and TβR-II-expressing 4T1 cells after PF-562271 treatment. These data point to an important role for FAK in regulating the composition and behavior of breast cancer stroma, particularly the recruitment of bone marrow-derived and other systemic immune cells whose presence is critical for mammary tumorigenesis [49]. To this end, we show a drastic reduction in tumor-infiltrating macrophages with FAK inhibition. Although a full characterization of the role for FAK in governing mammary stromal function clearly is warranted and currently is ongoing in our laboratory, the data presented here undoubtedly identify a novel tumor microenvironmental function for FAK that has yet to be fully appreciated.

## Conclusions

In summary, we demonstrate that FAK is activated upon TGF-β-mediated induction of EMT in a manner that requires β_3 _integrin and Src, and that the PTK activity of FAK is required for the physical linkage between β_3 _integrin and TβR-II, thereby generating the formation of oncogenic TGF-β signaling complexes. Indeed, our findings establish FAK as an essential player that facilitates the oncogenic conversion of TGF-β in developing and progressing mammary tumors, leading to their acquisition of invasive and metastatic phenotypes in response to TGF-β. Finally, we provide compelling evidence that inhibiting the PTK activity of FAK or its expression is sufficient to reduce the overall metastatic burden of highly aggressive breast cancers, and more specifically, that amplified TGF-β signaling in these same tumors is capable of driving the earliest steps of primary tumor metastasis, processes that are critically dependent on FAK.

## Abbreviations

E-cad: epithelial cadherin; EGF: epidermal growth factor; EMT: epithelial-mesenchymal transition; FAK: focal adhesion kinase; FBS: fetal bovine serum; MAPK: mitogen-activated protein kinase; MEC: mammary epithelial cell; p130Cas: Crk-associated substrate; PAI-1: plasminogen activator hnhibitor-1; pMSCV: plasmid murine stem cell virus; PTK: protein tyrosine kinase; shRNA: short hairpin RNA; TβR-I: TGF-β receptor type I; TβR-II: TGF-β receptor type II; TGF: transforming growth factor; WT: wild type.

## Competing interests

The authors declare that they have no competing interests.

## Authors' contributions

MKW participated in designing the study, acquiring the data, interpreting the data, analyzing the data, and preparing the manuscript. WPS participated in designing the study, interpreting the data, and preparing the manuscript.

## Supplementary Material

Additional file 1 A Word file containing a table listing the application and sequences of the various oligonucleotides used in the study Click here for file

Additional file 2 A TIFF file containing a figure showing that FAK deficiency decreases the growth rate and inhibits cytostasis mediated by TGF-β in normal MECs. **(a) **Control (*i.e*., scram) and FAK-deficient (shFAK) NMuMG and 4T1 cells were cultured in full growth media for 48 hours before addition of [^3^H]thymidine. Afterward, the extent of [^3^H]thymidine incorporation into cellular DNA was determined with scintillation counting, which showed that FAK deficiency significantly decreased the basal growth rate of NMuMG but not 4T1 cells. Data are expressed as the mean (± SEM) rates of DNA synthesis of each cell line relative to the corresponding controls observed in three independent experiments (*P < 0.05). **(b) **Control (*i.e*., scram) and FAK-deficient NMuMG (squares) and 4T1 (circles) cells were incubated for 48 hours in the absence or in the presence of increasing concentrations of TGF-β_1_, as indicated. Incorporation of [^3^H]thymidine into cellular DNA was determined as described earlier. Data are expressed as the mean (± SEM) rates of DNA synthesis relative to untreated controls observed in three independent experiments (*P < 0.05). Click here for file

Additional file 3 A JPEG file containing a figure characterizing the use of small-molecule inhibitors of FAK in NMuMG and 4T1 cells. **(a) **NMuMG cells were stimulated for 18 hours with TGF-β_1 _in the presence of the indicated concentrations of the FAK inhibitor PF-573228 (PF-228). Cells were subsequently lysed, and FAK autophosphorylation at Y397 was assayed. β-Actin serves as a loading control. **(b) **NMuMG cells were serum deprived for 4 hours in the absence or presence of the FAK inhibitor PF271 or PF228 (1 μ*M*), and subsequently were stimulated with TGF-β_1 _(5 ng/ml) for 30 minutes. Cells were lysed and assayed for FAK autophosphorylation at Y397. The membrane was stripped and reprobed for total FAK (tFAK) as a loading control. **(c) **Bright-field images of NMuMG cells under unstimulated (NS) conditions or those treated with TGF-β_1 _(5 ng/ml), PF-573228 (1 μ*M*), or both agents for 24 hours. **(d) **4T1 cells were incubated in the presence of increasing concentrations of the FAK inhibitor PF-562271 for 18 hours, at which point, the cells were lysed, and FAK autophosphorylation at Y397 was assayed. Membranes were stripped and reprobed for total FAK (tFAK) and β-actin as loading controls. **(e) **Mice bearing primary fat pad 4T1 tumors were left untreated (NS) or treated with PF-562271 (PF-271; 50 mg/kg/day). Four hours after the final treatment, the tumors were excised, and histologic sections were stained with antibodies specific for FAK autophosphorylation at Y397. Click here for file

Additional file 4 A TIFF file containing a figure showing that luminescent, metastatic 4T1 cells retain FAK knockdown *in vivo*, and that FAK knockdown has no effect on pulmonary invasion with tail injection. **(a) **Luminescent imaging of serially diluted 4T1 cells initially engineered to express firefly luciferase and subsequently scrambled (*i.e*., scram) or FAK-specific (shFAK) shRNAs. Data show that both 4T1 cell lines expressed identical quantities of luciferase. **(b) **Immunoblotting control (*i.e*., scram) and FAK-deficient (shFAK) 4T1 cells before their inoculation (pre-engraftment) into the mammary fat pads of Balb/C mice showed that FAK deficiency was maintained in metastatic 4T1 lesions. Subsequent to isolation from lungs of tumor-bearing mice, cells were cultured in the presence of Zeocin (antibiotic selection of firefly luciferase) (Metastatic) and similarly assayed for FAK expression. Immunoblotting for β-actin was performed as a loading control. **(c) **Control (*i.e*., scram) and FAK-depleted (shFAK) 4T1 cells expressing firefly luciferase were injected into the lateral tail vein of Balb/C mice (1 × 10^5 ^cells/mouse), and pulmonary invasion was measured at the indicated time points after injection. Data are expressed as the mean (± SEM; n = 10) area flux measurements normalized to the initial pulmonary flux measurement taken upon injection (T0) to correct for injection efficiency. Click here for file
