# Supplementary material for: Therapeutic targeting of the focal adhesion complex prevents oncogenic TGF-β signaling and metastasis
Source: Breast Cancer Res. 2009 Sep 9;11(5):R68. doi: 10.1186/bcr2360 (PMC2790843; doi:10.1186/bcr2360)
Supplement: Additional file 1 — A Word file containing a table listing the application and sequences of the various oligonucleotides used in the study [file bcr2360-S1.DOC]

| **Gene Target** | **Application** | **Sequence (5' to 3')** |
| --- | --- | --- |
| E-Cadherin | PCR-Sense | 5’-CCCTACATACACTCTGGTGGTTCA |
| E-Cadherin | PCR-Antisense | 5’-GGCATCATCATCGGTCACTTTG |
| PAI-1 | PCR-Sense | 5’-GGTGAAACAGGTGGACTTCTCA |
| PAI-1 | PCR-Antisense | 5’-GCATTCACCAGCACCAGGCGTG |
| Cox2 | PCR-Sense | 5’ -TGGGGTGATGA GCAACTATTCC |
| Cox2 | PCR-Antisense | 5’ -AGGCAATGCGGTTCTGATACTG |
| CK19 | PCR-Sense | 5' -TTGGGTCAGGGGGTGTTTTC |
| CK19 | PCR-Antisense | 5' -TTCTCATTGCCAGACAGCAGC |
| MMP9 | PCR-Sense | 5' -AGACCTGAAAACCTCCAACCTCAC |
| MMP9 | PCR-Antisense | 5' -TGTTATGATGGTCCCACTTGAGGC |
| Murine Beta3 Integrin | PCR-Sense | 5' -GTCCGCTACAAAGGGGAGAT |
| Murine Beta3 Integrin | PCR-Antisense | 5' -TAGCCAGTCCAGTCCGAGTC |
| FAK | PCR-Sense | 5'- GTGTAAAATTGGGAGACT |
| FAK | PCR-Antisense | 5'- GTAGCCTGTCTTCTGGAT |
| GAPDH | PCR-Sense | 5’-CAACTTTGGCATTGTGGAAGGGCTC |
| GAPDH | PCR-Antisense | 5’-GCAGGGATGATGTTCTGGGCAGC |
| Murine FAK | shRNA-Sense | 5’- GAATGGCAGCTGCTTATCTTtcaagag AAGATAAGCAGCTGCCATTCtttttt |
| Murine FAK | shRNA-Antisense | 5’- tcgagaaaaaaGAATGGCAGCTGCTTATCTTctcttga AAGATAAGCAGCTGCCATTCa |
