# Supplementary figures and images for: Therapeutic targeting of the focal adhesion complex prevents oncogenic TGF-β signaling and metastasis
Source: Breast Cancer Res. 2009 Sep 9;11(5):R68. doi: 10.1186/bcr2360 (PMC2790843; doi:10.1186/bcr2360)

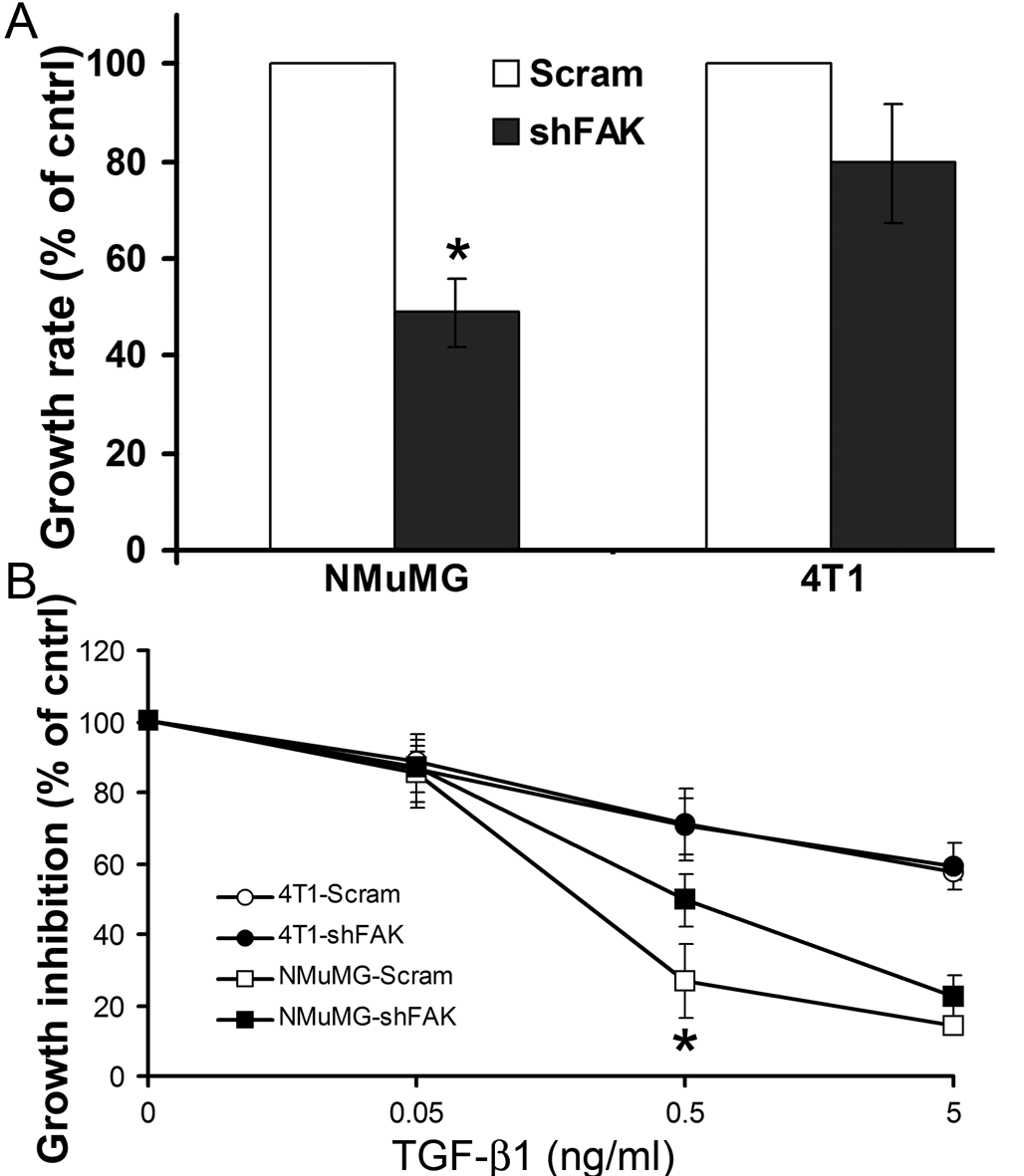

Supplement: Additional file 2 — A TIFF file containing a figure showing that FAK deficiency decreases the growth rate and inhibits cytostasis mediated by TGF-β in normal MECs. (a) Control (i.e., scram) and FAK-deficient (shFAK) NMuMG and 4T1 cells were cultured in full growth media for 48 hours before addition of [3H]thymidine. Afterward, the extent of [3H]thymidine incorporation into cellular DNA was determined with scintillation counting, which showed that FAK deficiency significantly decreased the basal growth rate of NMuMG but not 4T1 cells. Data are expressed as the mean (± SEM) rates of DNA synthesis of each cell line relative to the corresponding controls observed in three independent experiments (*P < 0.05). (b) Control (i.e., scram) and FAK-deficient NMuMG (squares) and 4T1 (circles) cells were incubated for 48 hours in the absence or in the presence of increasing concentrations of TGF-β1, as indicated. Incorporation of [3H]thymidine into cellular DNA was determined as described earlier. Data are expressed as the mean (± SEM) rates of DNA synthesis relative to untreated controls observed in three independent experiments (*P < 0.05). [file bcr2360-S2.TIFF]

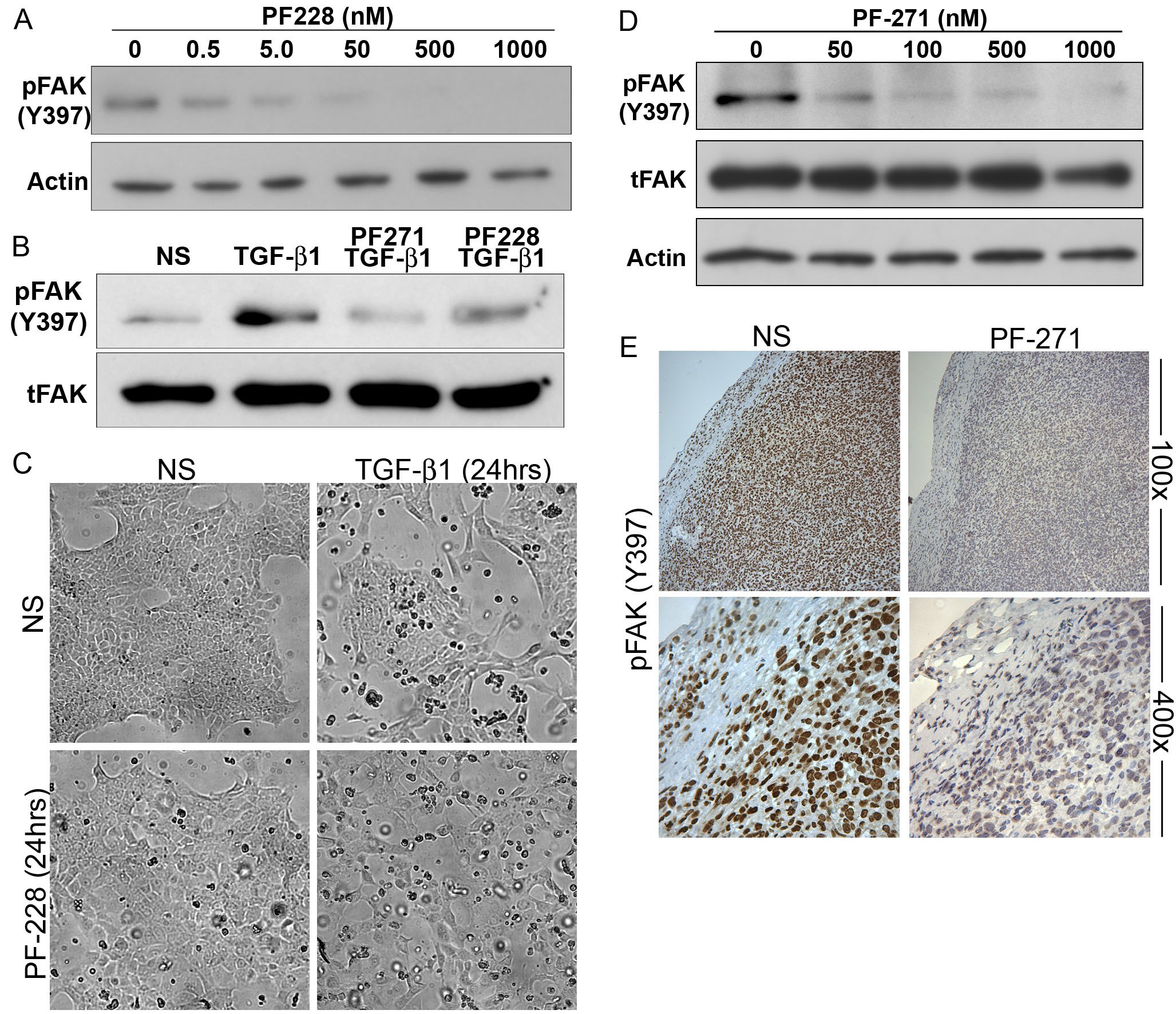

Supplement: Additional file 3 — A JPEG file containing a figure characterizing the use of small-molecule inhibitors of FAK in NMuMG and 4T1 cells. (a) NMuMG cells were stimulated for 18 hours with TGF-β1 in the presence of the indicated concentrations of the FAK inhibitor PF-573228 (PF-228). Cells were subsequently lysed, and FAK autophosphorylation at Y397 was assayed. β-Actin serves as a loading control. (b) NMuMG cells were serum deprived for 4 hours in the absence or presence of the FAK inhibitor PF271 or PF228 (1 μM), and subsequently were stimulated with TGF-β1 (5 ng/ml) for 30 minutes. Cells were lysed and assayed for FAK autophosphorylation at Y397. The membrane was stripped and reprobed for total FAK (tFAK) as a loading control. (c) Bright-field images of NMuMG cells under unstimulated (NS) conditions or those treated with TGF-β1 (5 ng/ml), PF-573228 (1 μM), or both agents for 24 hours. (d) 4T1 cells were incubated in the presence of increasing concentrations of the FAK inhibitor PF-562271 for 18 hours, at which point, the cells were lysed, and FAK autophosphorylation at Y397 was assayed. Membranes were stripped and reprobed for total FAK (tFAK) and β-actin as loading controls. (e) Mice bearing primary fat pad 4T1 tumors were left untreated (NS) or treated with PF-562271 (PF-271; 50 mg/kg/day). Four hours after the final treatment, the tumors were excised, and histologic sections were stained with antibodies specific for FAK autophosphorylation at Y397. [file bcr2360-S3.JPEG]

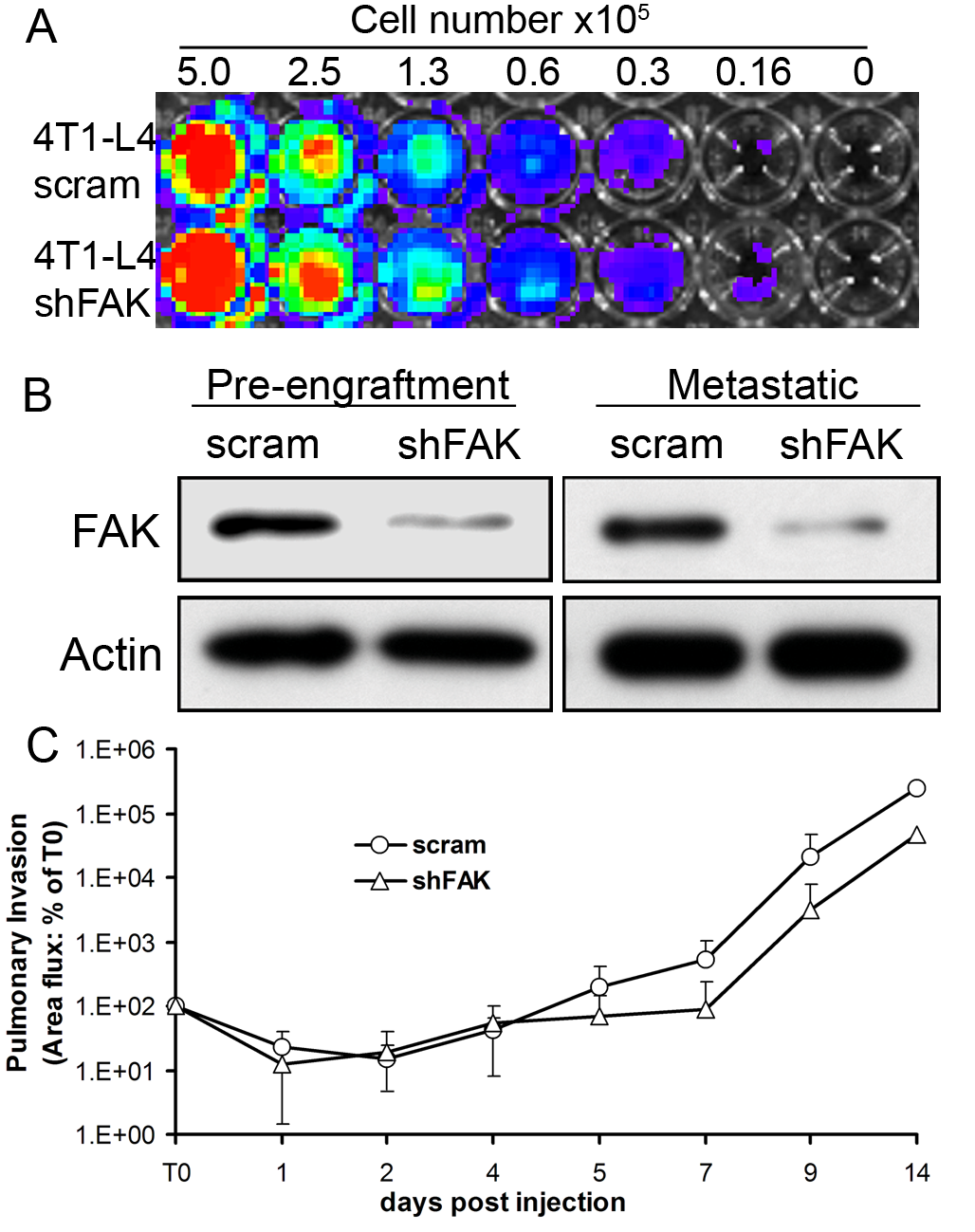

Supplement: Additional file 4 — A TIFF file containing a figure showing that luminescent, metastatic 4T1 cells retain FAK knockdown in vivo, and that FAK knockdown has no effect on pulmonary invasion with tail injection. (a) Luminescent imaging of serially diluted 4T1 cells initially engineered to express firefly luciferase and subsequently scrambled (i.e., scram) or FAK-specific (shFAK) shRNAs. Data show that both 4T1 cell lines expressed identical quantities of luciferase. (b) Immunoblotting control (i.e., scram) and FAK-deficient (shFAK) 4T1 cells before their inoculation (pre-engraftment) into the mammary fat pads of Balb/C mice showed that FAK deficiency was maintained in metastatic 4T1 lesions. Subsequent to isolation from lungs of tumor-bearing mice, cells were cultured in the presence of Zeocin (antibiotic selection of firefly luciferase) (Metastatic) and similarly assayed for FAK expression. Immunoblotting for β-actin was performed as a loading control. (c) Control (i.e., scram) and FAK-depleted (shFAK) 4T1 cells expressing firefly luciferase were injected into the lateral tail vein of Balb/C mice (1 × 10^5^ cells/mouse), and pulmonary invasion was measured at the indicated time points after injection. Data are expressed as the mean (± SEM; n = 10) area flux measurements normalized to the initial pulmonary flux measurement taken upon injection (T0) to correct for injection efficiency. [file bcr2360-S4.TIFF]
